# Effects of the histone deacetylase inhibitor valproic acid in combination with fear-memory retrieval before exposure therapy for spider phobia: A randomized controlled trial

**DOI:** 10.1017/S0033291725101475

**Published:** 2025-09-12

**Authors:** Nathalie Schicktanz, Frieder Dechent, Carlo Andreas Huber, Anja Zimmer, Galya Clara Iseli, Jeanne Howald, Maya Thalia Schenker, Johannes Gräff, Undine Lang, Dominique J. F. de Quervain, Dorothée Bentz

**Affiliations:** 1 Division of Cognitive Neuroscience, University of Basel, Basel, Switzerland; 2 University Psychiatric Clinics Basel, Basel, Switzerland; 3 Research Cluster Molecular and Cognitive Neurosciences, Department of Biomedicine, University of Basel, Basel, Switzerland; 4 École Polytechnique Fédérale Lausanne (EPFL), Laboratory of Neuroepigenetics, Brain Mind Institute, School of Life Sciences, Lausanne, Switzerland; 5 École Polytechnique Fédérale Lausanne (EPFL), Synapsy Center for Neuroscience and Mental Health Research, School of Life Sciences, Lausanne, Switzerland

**Keywords:** exposure therapy, fear-memory retrieval, histone deacetylase inhibitor (HDACi), phobic disorders, remote fear-memory attenuation

## Abstract

**Background:**

Return of fear after successful exposure therapy for a phobia is a common clinical challenge. A previous study on mice demonstrated that the persistent attenuation of remote fear memories can be achieved by combining histone deacetylase inhibitors (HDACis) with fear-memory retrieval prior to extinction training.

**Methods:**

To evaluate the translational potential of this approach, we conducted a randomized, double-blind, placebo-controlled trial. Forty-eight individuals with DSM-IV spider phobia received either HDACi valproic acid (VPA, 500 mg) or a placebo prior to the retrieval of fear memory, followed by exposure therapy in virtual reality.

**Results:**

No significant group difference was found in terms of behavioral change on the behavioral approach test at 3 months follow-up and baseline (primary outcome). However, the VPA group displayed significantly reduced fear in two self-report questionnaires related to spider phobia (Fear of Spiders Questionnaire; Spider Phobia Beliefs Questionnaire) as compared to the placebo group. No group differences were observed for psychophysiological indicators of fear.

**Conclusions:**

The favorable impact of a single administration of VPA in combination with fear-memory retrieval prior to exposure therapy suggests that it might be an effective way to enhance symptom improvement at the subjective level in the treatment of phobias. Further studies need to investigate the conditions under which an improvement on the psychophysiological and behavioral levels can be achieved as well.

## Introduction

Specific phobias are a common mental disorder with an estimated lifetime prevalence of 12.5% (Michael, Zetsche, & Margraf, 2007). The symptomatology is characterized by excessive fear and avoidance behavior cued to specific objects or situations (American Psychiatric Association, 2013). Exposure therapy is the state-of-the-art treatment approach for specific phobias (Chambless & Ollendick, 2001). The hypothesized mode of action for fear reduction in exposure therapy is extinction (Hamlett, Foa, & Brown, 2023), a process that establishes an alternative non-fear memory to compete with and suppress the original fear memory (Milad & Quirk, 2012). As a consequence, the original fear memory – although no longer expressed – remains intact and can return over time, upon context renewal, or when the original fearful object or situation is unexpectedly encountered (Bouton, 1993, 2004; Bouton & Bolles, 1979; Rescorla, 2004; Rescorla & Heth, 1975). The return of fear accordingly remains a common challenge in clinical practice, despite the success of fear reduction in short-term assessments following standard exposure therapy (Bentz, Michael, De Quervain, & Wilhelm, 2010; Craske & Mystkowski, 2006), so developing novel approaches to improve the stability of treatment results is of importance.

Targeting the learning and memory processes fundamental to exposure therapy represents a viable strategy. In this context, glucocorticoids with their memory-modulating properties have shown promising results in enhancing treatment success in various anxiety disorders including specific phobias, potentially by weakening aversive-memory retrieval and strengthening extinction memories (Astill Wright et al., 2019; De Quervain et al., 2011; De Quervain, Schwabe, & Roozendaal, 2017). An alternative approach is to target the fear-memory reconsolidation process, especially if the goal is not only to enhance the efficacy of exposure therapy in reducing symptoms but also to increase the stability of treatment outcomes. Fear-memory reconsolidation refers to the process by which a previously consolidated fear memory undergoes a brief phase of instability after retrieval (often referred to as memory reactivation) before entering a new phase of stabilization. This process involves the synthesis of new proteins and RNA to stabilize the retrieved memory, presenting a window of opportunity to modify the existing memory permanently (Alberini, 2011; Nader, Schafe, & Le Doux, 2000; Przybyslawski & Sara, 1997). Whether the original or a modified memory is subsequently reconsolidated depends on various factors (for an overview, see Auber, Tedesco, Jones, Monfils, & Chiamulera, 2013; Kindt & Elsey, 2023). These so-called boundary conditions include factors referring not only to the kind of retrieval cue and its timing but also to the fear memory itself and the approach used to target reconsolidation.

There are different approaches in use to destabilize and modify existing memories that can be divided into purely pharmacological or behavioral approaches or a combination of both. Pharmacological approaches involve administering substances that disrupt the reconsolidation process after retrieval (e.g., the protein synthesis inhibitor anisomycin used in the seminal animal study by Nader et al. [2000] or propranolol in humans [Kindt, Soeter, & Vervliet, 2009]). Behavioral approaches refer to retrieval followed by the provision of new information. This approach was introduced by the retrieval-extinction paradigm in which extinction follows a short retrieval of a formerly acquired fear memory (animals: Monfils, Cowansage, Klann, & LeDoux, 2009; humans: Schiller et al., 2010). Both pharmacological and behavioral approaches have been found to successfully modify experimentally established fear memories in animals and humans using fear conditioning as the paradigm (Bentz & Schiller, 2015; Schwabe, Nader, & Pruessner, 2014). The last decade has seen a large number of translational studies testing immediate symptom reduction based on either a behavioral or a pharmacological paradigm and the sustainability of these effects. For example, Soeter and Kindt (2015) describe how the application of propranolol after fear-memory retrieval in patients with spider phobia resulted in a greater symptom improvement compared to a placebo that persisted for at least 1 year after the procedure. However, the benefits of the propranolol reconsolidation paradigm have not been equally observed in all translational studies in anxiety disorders, including specific phobias (Elsey et al., 2020; Steenen et al., 2016). For the behavioral approach, the majority of studies have looked at the enhancement of symptom reduction through retrieval followed by extinction-based therapy compared to standard exposure therapy. For this approach too, the findings are not unequivocal, as some studies with participants suffering from specific fears (e.g., spiders, flying) have found improvements that lasted for up to 6 months (e.g., Björkstrand et al., 2017; Maples-Keller et al., 2017), while others investigating the same fears found no effects (Shiban, Brütting, Pauli, & Mühlberger, 2015).

Taken together, attempts to target reconsolidation behaviorally or pharmacologically have not been consistently effective in patients with anxiety disorders, including specific phobias, whose symptoms are based on long-lasting fear memory. In this context, a study in mice showed that 1-day-old fear memories differ from remote fear memories acquired in the same classical-conditioning paradigm. This difference is not apparent in short-term outcomes (freezing) but becomes evident in long-term outcomes at a 30-day follow-up. Notably, only the remote-memory group exhibited a return of fear (Gräff et al., 2014). This finding is highly relevant, as efforts have primarily focused on translating findings from recent memories in the realm of reconsolidation research into clinical applications. The aforementioned study showed that whereas the recall of recent memories induced a brief period of hippocampal neuroplasticity, partly mediated by the S-nitrosylation of HDAC2 and histone acetylation, the recall of remote memories did not induce such neuroplasticity. Crucially, the same study showed that administering an HDAC2-targeting histone deacetylase inhibitor (HDACi) during the reconsolidation process effectively and persistently attenuated remote memories in a retrieval-extinction protocol (Gräff et al., 2014). The application of the HDACi was found to epigenetically prime the expression of neuroplasticity-related genes, enabling long-term modifications of remote memories.

Consequently, the application of HDACis in combination with the retrieval of a fear memory prior to extinction-based therapy may offer a novel therapeutic strategy for the persistent reduction of remote aversive memories. In the present study, valproic acid (VPA), a known HDACi, was combined with the retrieval of remote fear memories in individuals with spider phobia undergoing exposure therapy in virtual reality (VR). The purpose of this study was to evaluate whether VPA in combination with fear-memory retrieval enhances exposure therapy for phobias.

## Experimental procedures

### Study design and participants

**Figure 1.**
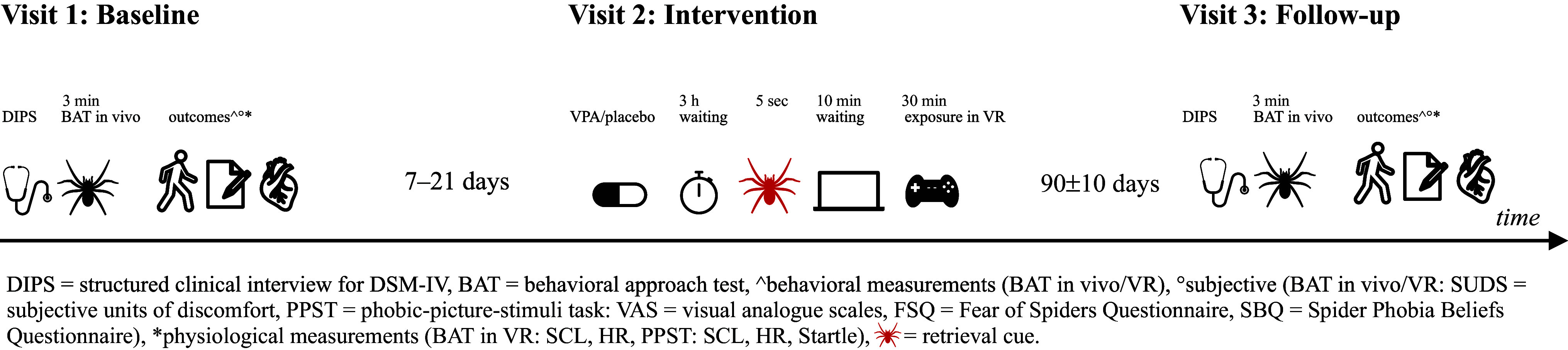
Study schedule. This figure illustrates the sequence of the study tasks for obtaining the primary and secondary outcome measurements at baseline (visit 1) and follow-up (visit 3), as well as the sequence of the intervention during visit 2.

We performed a double-blind, parallel-group (intervention versus control), randomized controlled trial (RCT) comparing the effect of the combination of VPA + retrieval with the effect of a placebo + retrieval prior to exposure therapy for a specific phobia (animal type: spiders). The RCT comprised three visits. For an overview of the study procedure and tasks, see Figure 1. Healthy participants aged 18–40 years and diagnosed with a DSM-IV specific phobia (animal type: spiders) were recruited. Because the prevalence of specific phobia (animal type: spider) is estimated to be 4–5 times higher in females compared to males (Fredrikson, Annas, Fischer, & Wik, 1996; Oosterink, De Jongh, & Hoogstraten, 2009) we allowed for a recruitment of predominantly female participants. To be eligible for the study, participants were required to have a score of 1–7 points on a real-life behavioral approach test (BAT in vivo) to ensure their willingness to subject themselves to exposure and to prevent ceiling effects after intervention. The study protocol of the clinical trial (ClinicalTrials.gov, identifier: NCT02789813), including the definition of the primary and secondary outcomes and the plan for the statistical analysis, was approved by the Ethics Committee of Northwest and Central Switzerland (EKNZ) before the start of the study. The entire study was performed in accordance with the Declaration of Helsinki. All participants gave written informed consent to trial participation. Participants received a compensation of CHF 250 for participating in the trial.

### Randomization and masking

Participants were randomly assigned by the experimenter to treatment groups (VPA + retrieval or placebo + retrieval), with stratification by sex. The randomization lists and study medication were prepared by the hospital pharmacy at the University Hospital of Basel in Switzerland. Block randomization in groups of four was used. Each block included two allocations for each treatment condition. Visually identical syrups containing VPA or placebo, filled in 20 ml glass bottles with identical labels, were provided. These measures ensured the concealment of assignments from the enrolling physician, experimenter, and participants, maintaining a double-blind approach regarding VPA administration. Additionally, the personnel processing psychological and psychophysiological data remained unaware of participant group assignments.

### Study tasks

#### Retrieval

Participants were shown a virtual spider for 5 seconds in a virtual room (see Supplementary Figure SF2) to initiate retrieval of the fear memory. This was done 10 minutes prior to exposure therapy to target the reconsolidation window (Schiller et al., 2010). Afterward, participants were asked to indicate the fear they experienced via a Subjective Units of Distress Scale (SUDS) (0 = no fear, 10 = maximum fear) (Wolpe, 1969). Electrodermal activity (EDA) and electrocardiography (ECG) were measured during the 5 seconds of the presentation of the virtual spider.

#### Exposure in VR

For exposure therapy, we used the 30-minute hierarchical VR-exposure protocol employed in the study by Shiban et al. (2015). A brief exposure protocol was chosen to maintain sufficient room for detecting a potential treatment effect of the combination of VPA + retrieval. During each of the ten 3-minute scenes in VR, participants rated their fear on the SUDS at the beginning and end of each scene. Minimal verbal support was given by the experimenter, who also checked for simulation sickness and recorded the SUDS measurements. Psychophysiological measurements (EDA, ECG) were continuously taken during exposure.

#### BAT in vivo

We used a BAT to measure approach behavior to a living house spider (*Tegenaria atrica*, size: approx. 5 cm). This BAT in vivo corresponded to the one used by Lass-Hennemann and Michael (2014). Before starting the BAT, participants rated their expected fear during the BAT using a SUDS (0 = no fear, 10 = maximum fear imaginable). The BAT assessed participants’ willingness to approach a spider in a sealed transparent container following 12 graded steps (cumulative points per step in brackets): refuses to enter the room (0), stops at 5 m (1), 4 m (2), 3 m (3), 2 m (4), 1 m (5), near the container (6), touches the container (7), removes the lid (8), puts a hand inside (9), touches the spider (10), holds the spider (<20s = 11, ≥20s = 12). The task ended when the participant could not proceed, completed the last step, or after 3 minutes. At the final position reached, participants rated their actual fear with a SUDS.

#### BAT in VR

We used a BAT to measure approach behavior to a VR spider. Participants had 3 minutes to approach the virtual spider. Before the 3 minute period began, participants’ position was fixed with the virtual spider in their field of vision for 15 seconds to record SUDS and psychophysiological fear indicators (EDA, ECG) in a standardized manner. Approach behavior was determined by means of virtual distance to the spider at the end of the BAT in VR.

#### Phobic-picture-stimuli task (PPST)

The phobic-picture-stimuli task (PPST) was designed to obtain subjective and psychophysiological reactions to fear-related (spider), negative (snake), and neutral stimuli. Half of the presented pictures in each category were combined with startle probes (white noise, 100 dB). The duration of the main phase of the PPST was approximately 20 minutes.

### Outcomes

Our primary outcome was the change in approach behavior as measured by the difference in the BAT in vivo scores between visit 1 (baseline) and visit 3 (follow-up) (BAT difference score). The scores ranged from 0 to 12 (higher scores indicate higher approach behavior).

Our secondary outcomes were the differences between visit 1 (baseline) and visit 3 (follow-up) (difference score) in the following additional behavioral measures: (1) the BAT in VR and, subjective measures: (2) the SUDS after BAT in vivo, (3) the SUDS after BAT in VR, (4) the Fear of Spiders Questionnaire (FSQ; sum score ranges from 0 to 108) (Szymanski & O’Donohue, 1995), (5) the Spider Phobia Beliefs Questionnaire (SBQ; mean sum score 0–100) (Arntz, Lavy, Van Den Berg, & Van Rijsoort, 1993), (6) the DSM-IV impairment score, (7) DSM-IV distress score, (8) the State–Trait Anxiety Inventory state version (STAI-S) (Spielberger, Gorsuch, Lushene, Vagg, & Jacobs, 1970), (9) the valence ratings of spider pictures, (10) the arousal ratings of spider pictures, (11) the anxiety ratings of spider pictures, and psychophysiological measures: (12) heart rate (HR) BAT in VR, (13) skin conductance levels (SCL) BAT in VR, (14) HR while looking at spider pictures, (15) SCL while looking at spider pictures, (16) startle reactions to spider pictures (for details on the secondary outcomes, see the supplementary information).

### Statistical analyses

We conducted a per-protocol analysis using R Studio, version 4.2.3 (R Core Team, 2021). Linear models were applied to assess the impact of our interventions. These models incorporated ANOVA (type II sums of squares) and were used with dependent variables representing the differences in the measurements at visit 3 and visit 1. The covariates included age and sex, while group allocation served as an independent variable. Effect sizes were calculated using Cohen’s *d.* The model assumptions were checked using the Performance package in R (Lüdecke, Ben-Shachar, Patil, Waggoner, & Makowski, 2021) and confirmed for the main analysis (for additional analysis, see the supplementary information). The significance threshold was set at *p* < 0.05 for the primary outcomes and *p* < 0.003125 (Bonferroni correction for 16 independent tests) for the secondary outcomes. We aimed to detect large drug effects (*d* = 0.8) with 80% power and α = 0.05. A power analysis (G*Power 3.1) indicated 25 participants per group was the minimum group size to measure statistically relevant effects.

### Deviations from the initial protocol

**Figure 2.**
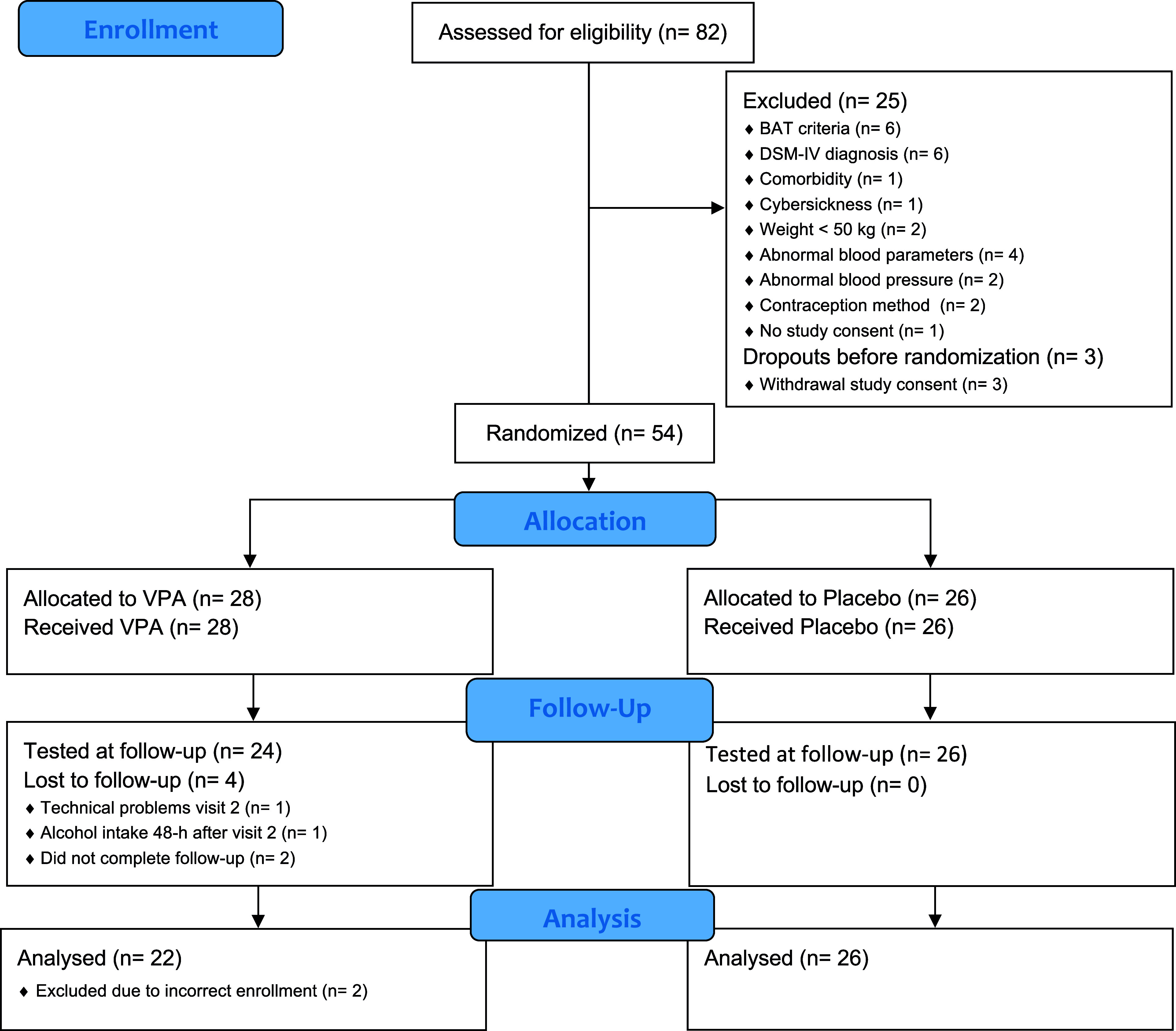
CONSORT flow chart. The flow chart illustrates the participants’ progression through the study, including enrollment (visit 1), allocation (visit 2), follow-up (visit 3), and analysis for those recruited for the VPA + retrieval and placebo + retrieval groups. BAT = behavioral approach test; VPA = valproic acid.

The approved study protocol foresaw four experimental groups with a total of 100 participants: intervention (VPA + retrieval), control I (placebo + retrieval), control II (VPA + no retrieval), and control III (placebo + no retrieval). To ensure that we would be able to answer our main question on the impact of VPA + retrieval on exposure therapy for phobias despite potential recruitment difficulties, we first recruited participants for the intervention and control I. Due to slow recruitment, we conducted an interim analysis with 48 participants. The study was terminated after the interim analysis on May 24, 2018, as it did not support our primary hypothesis that VPA + retrieval would lead to greater approach behavior as measured in the BAT in vivo compared to placebo + retrieval. The data from the participants recruited for control II and III were not analyzed and are not shown in the flow chart (see Figure 2).

## Results

### Participants’ characteristics and study flow

Eighty-two individuals were screened for trial participation. Fifty-four participants were randomized (28 received VPA, 26 received a placebo), 50 participants were tested at visit 3, and 48 participants were included in the analysis. For full information about the participants’ study flow see Figure 2; for the participants’ characteristics see Table 1.

**Table 1.** Participants’ characteristics

	Placebo	VPA
Sex (female/male)	23/3	20/2
Comorbid specific phobia (y/n)	11/15	8/14
DSM-IV diagnoses (animal type: spider) at follow-up (y/n)	19/7	14/8
Age (*SD*)	24.42 (4.44)	24.41 (4.33)
Body mass index (*SD*)	24.05 (3.88)	23.31 (3.18)

### Retrieval

There was no significant group difference in the SUDS ratings after the retrieval procedure (*p* = 0.11, placebo: mean = 4.38, *SD* = 2.64, range 0–9; VPA: mean = 5.54, *SD* = 2.06, range 2–9). Furthermore, there was no significant group difference in the EDA and ECG measurements (all *p* > 0.37).

### Effect of VPA on primary and secondary outcomes

**Figure 3.**
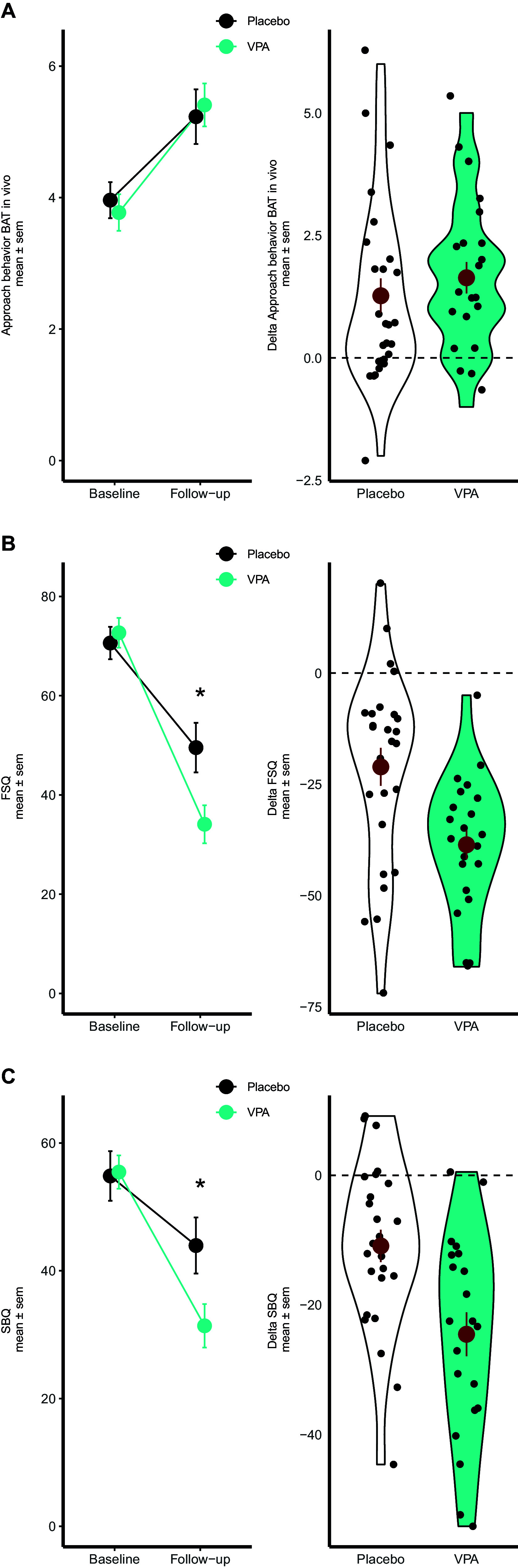
Primary and significant secondary outcomes at visit 1 (baseline) and visit 3 (follow-up). The black line represents the placebo + retrieval group, and the turquoise line represents the valproic acid + retrieval group. Displayed are the means and standard errors of the means. BAT in vivo = behavioral approach test in vivo (0–12), FSQ = Fear of Spiders Questionnaire (sum score ranges from 0 to 108), SBQ = Spider Phobia Beliefs Questionnaire (mean sum score ranges from 0 to 100). On the left side, the raw values of both visits are displayed. On the right side, the delta outcome variables between follow-up and baseline are displayed.

Group allocation did not have a statistically significant impact on the primary outcome, the difference in approach behavior as measured by the BAT in vivo (*F* (1, 44) = 0.56, *p* = 0.46, *d* = 0.23). As a robustness check, we additionally residualized the difference score in BAT on age and sex and compared the resulting residuals between treatment groups using a Mann–Whitney U test. This analysis also yielded a non-significant main effect of group (*U* = 233.5, *p* = 0.28, *r* = 0.16), supporting the findings from the linear model. For the secondary outcomes, Bonferroni-corrected significant differences were observed between the groups in two of the 16 secondary outcome measures, both of which were subjective measures queried by self-report questionnaires assessing the fear of spiders: FSQ (*F* (1, 44) = 9.78, *p* = 0.003, *d* = −0.95), and the SBQ (*F* (1, 43) = 11.09, *p* = 0.002, *d* = −1.02) (see Figure 3 and Table 2). These findings indicate a more pronounced reduction in the subjective experience of phobic fear in the VPA + retrieval group compared to the placebo + retrieval group. None of the other secondary outcomes showed a significant group-allocation difference after a Bonferroni correction (*p* ≥ 0.019; see the supplementary information).

**Table 2.** Descriptive statistics of primary and significant secondary outcomes, effect sizes, and *p* values

Outcome	Time	Group	n	Mean	Median	SD	Min	Max	Cohen’s *d*	p	Effect size
Approach behavior BAT in vivo	Baseline	Placebo	26	3.96	4.00	1.40	1.00	6.00			
	Baseline	VPA	22	3.77	3.50	1.31	1.00	6.00	−0.14		
	Follow-up	Placebo	26	5.23	5.00	2.12	1.00	8.00			
	Follow-up	VPA	22	5.41	5.00	1.53	2.00	8.00	0.10		
	Difference score	Placebo	26	1.27	1.00	1.80	−2.00	6.00			
	Difference score	VPA	22	1.64	1.50	1.50	−1.00	5.00	0.22	0.46	*d* = 0.23
FSQ	Baseline	Placebo	26	70.62	70.50	16.74	37.00	97.00			
	Baseline	VPA	22	72.68	71.00	14.08	45.00	99.00	0.13		
	Follow-up	Placebo	26	49.54	50.00	25.53	3.00	86.00			
	Follow-up	VPA	22	34.09	30.00	17.97	6.00	75.00	−0.69		
	Difference score	Placebo	26	−21.08	−14.00	21.74	−72.00	20.00			
	Difference score	VPA	22	−38.59	−36.50	15.34	−66.00	−5.00	−0.92	0.003	*d* = −0.95
SBQ	Baseline	Placebo	26	54.84	59.58	19.85	4.17	90.54			
	Baseline	VPA	21^a^	55.45	51.35	11.96	31.04	77.71	0.04		
	Follow-up	Placebo	26	43.94	47.92	22.38	0.83	80.42			
	Follow-up	VPA	22	31.38	31.88	16.00	10.63	71.88	−0.64		
	Difference score	Placebo	26	−10.89	−10.64	12.75	−44.63	9.17			
	Difference score	VPA	21^a^	−24.53	−22.50	15.49	−54.17	0.52	−0.97	0.002	*d* = −1.02

*Note:* VPA, valproic acid; FSQ, Fear of Spiders Questionnaire (sum score ranges from 0 to 108); SBQ, Spider Phobia Beliefs Questionnaire (mean sum score ranges from 0 to 100); *SD*, standard deviation; min, minimum; max, maximum; Cohen’s *d* refers to effect size calculated based on mean values, *p* refers to *p* value of main effect of group allocation in the used statistical model, i.e., difference between follow-up and baseline. Effect size refers to effect size of main effect of group allocation in the used statistical model, i.e., statistical model including age and sex as covariates (for more information, see the supplementary information).

a SBQ was not collected for one participant at baseline.

### Valproic acid in blood levels before and after study-medication intake at visit 2

Before study-medication intake, all the participants had undetectable levels of VPA in their blood (the laboratory sensitivity was ≥2.8 mg/l, values labeled by the laboratory with <2.8 mg/l were set to 0). Following the intake of the study medication, the blood levels for the placebo + retrieval group remained at zero (mean = 0, *SD* = 0). For the VPA group, there was a notable increase in blood levels (mean = 40.27, *SD* = 6.28, min = 25.20, max = 49.60, unit = mg/l). This increase is significantly different from zero (one-sample *t* test against zero, *t* (19) = 28.69, *p* < 2.2E-16).

### Participants’ perception of intake of VPA or placebo

There were no significant group differences in the participants’ perception of having received VPA or placebo when asked at visit 2 or visit 3 (*p* ≥ 0.18) (for more information, see the supplementary information).

### Adverse events

There were no significant group differences in the occurrence of adverse events at visit 2 after medication intake (*p* > 0.49). Eleven participants from the VPA + retrieval group reported 15 adverse events overall, while 14 participants from the placebo + retrieval group reported 23 adverse events at visit 2 after medication intake (see Supplementary Tables ST4 and ST5).

## Discussion

The present study found no significant differences in the primary outcome – approach behavior as measured in a real-life BAT conducted 3 months after the intervention – between the group that received VPA in combination with retrieval prior to exposure therapy versus the group that received a placebo in combination with retrieval prior to exposure therapy. However, noteworthy findings were seen in the secondary outcomes, where two out of the 16 outcomes displayed significant effects after a Bonferroni correction. Specifically, these two secondary outcomes revealed a significant reduction in subjective fear of spiders as assessed by the FSQ and the SBQ in the group that received VPA in combination with retrieval as compared to the group that received a placebo in combination with retrieval. This reduction was observed in addition to the effects of exposure therapy itself, which exhibited a medium effect size in the control group (generalized semipartial R2, FSQ: 0.22, SBQ: 0.11). The FSQ and SBQ were used to assess self-reported emotions and anticipated reactions in the presence of spiders, including cognitive aspects of these emotions. Importantly, there were no significant differences in the participants’ perceptions of whether they had received VPA or a placebo. This indicates that the subjective emotional and cognitive responses were not biased by the participants’ perceptions of their treatment allocation.

Beyond the VPA effects on fear as assessed with the two questionnaires, no additional effects were observed in the participants’ actual fear responses in the presence of a living spider or a virtual one, in their psychophysiological reactions, in DSM-IV impairment and distress criteria, or in their general state anxiety. Our primary and secondary outcomes assessed distinct aspects of phobic fear (behavioral, subjective *–* both cognitive and emotional, and psychophysiological), and our results may indicate that the proposed mechanism of action of the study medication did not affect all of these aspects similarly following a single dose of medication. LeDoux and Pine (2016) argue that responses to threats should be differentiated into behavioral and subjective components. They propose that two distinct brain circuits underlie: (1) behavioral responses, accompanied by physiological changes in the brain and body and (2) conscious feeling states, as reflected in self-reports of fear and anxiety. While behavioral and psychophysiological responses remained unaffected in our study, the findings for conscious feeling states were mixed. The self-report spider fear questionnaires (FSQ and SBQ) showed medication-related differences, whereas the SUDS ratings during the BAT did not. This discrepancy may reflect differences in what these measures capture: the FSQ and SBQ assess cognitive appraisals of anticipated fear in imagined scenarios, while SUDS ratings during the BAT reflect immediate, situation-specific emotional responses – essentially, in-the-moment fear in the presence of a feared stimulus. The questionnaires involve cognitive appraisal and imagination of hypothetical encounters with spiders, assessing beliefs, expectations, and anticipatory fear. In contrast, the SUDS ratings during the BAT reflect immediate, situation-bound emotional responses – essentially, in-the-moment fear in the presence of a feared stimulus. It is possible that the intervention influenced cognitive appraisals of fear more readily than acute emotional or behavioral responses. In line with this, other studies implementing a single exposure therapy session also showed an effect on self-report questionnaires but not on approach behavior as measured in a BAT in vivo (Bentz et al., 2021; Zimmer et al., 2021). It thus remains to be investigated whether different dosages or repeated administrations of VPA, in combination with exposure therapy, might affect not only cognitive appraisals of anticipated fear, but also other aspects of phobic fear.

During the exposure sessions, the VPA and placebo groups did not exhibit significant differences in terms of SUDS, SCL, and HR at the beginning and end of each exposure scene level and when comparing the beginning and end of the complete exposure session. This suggests that VPA had no effect on extinction learning. A previous human study (Kuriyama, Honma, Soshi, Fujii, & Kim, 2011) also found that 400 mg of VPA had no effect on extinction learning, as quantified by SCL in a 3-day fear-learning paradigm. Furthermore, in that study, VPA had no effect on recall after extinction, but it did show a reduced reinstatement of the conditioned association. Moreover, a study in mice showed that VPA enhanced memory consolidation for both the acquisition and the extinction of cued fear (Bredy & Barad, 2008). It is thus possible that the beneficial effects of VPA on subjective fear in the present study were related to an enhancement of the consolidation of extinction memories.

Prior studies have established VPA as a strong HDACi (Bredy & Barad, 2008). However, it is important to note that our study did not specifically examine changes in histone acetylation. Consequently, we lack information as to whether VPA altered the fear memory to transition into a transcriptionally more permissive chromatin state, as has been previously shown in mice (Bredy & Barad, 2008; Gräff et al., 2014). If this occurred, it could have potentially served as a molecular mechanism behind the observed effects in the reduction of fear in the self-report questionnaires. Furthermore, VPA does not target class I HDACs with high affinity (Burns & Gräff, 2021) like HDAC2, which has been identified to mediate memory updating during extinction training in mice (Gräff et al., 2014). Thus, exploring whether alterations in histone acetylation and/or HDAC2 activity or whether more specific HDAC2-targeting HDACis mediate fear reduction following intervention and treatment could provide valuable insights for more refined studies in the future.

Limitations: Despite the large number of secondary outcome measures and the need for corrections for multiple comparisons, our study was based on a relatively small clinical sample size and was only sufficiently powered for our primary outcome. Furthermore, we did not test VPA and placebo groups without retrieval prior to exposure therapy as we had originally planned because it surpassed our resources due to slow recruitment. We therefore do not know if the same results would have been achieved without prior retrieval, based on the enhancing effect of VPA on the consolidation of extinction memories. Furthermore, one might argue that even with the inclusion of a control condition without retrieval, we would not be able to infer that our results are due to targeting the reconsolidation window, as the mere presence of the retrieval cue may have been sufficient to pharmacologically augment the extinction process. We are thus not able to verify all the criteria that have been suggested to be necessary for inferring that reconsolidation was involved in the favorable effects of our intervention (for an overview, see Schroyens, Beckers, & Luyten, 2023). Nevertheless, a study by Shiban et al. (2015) used a similar translation of the retrieval-extinction paradigm into a clinical application without any additional pharmacological interventions and included such a control group, and it found no improvement from retrieval before extinction-based therapy compared to standard extinction-based therapy without prior retrieval.

To conclude, our results indicate that a single administration of VPA before the retrieval of fear memories prior to exposure therapy led to a significant reduction in subjective measures of phobic fear levels 3 months later in comparison to the effects observed with no active substance and fear-memory retrieval prior to exposure therapy. This finding implies that the addition of VPA may hold promise as an additional therapeutic approach for enhancing treatment outcomes in individuals with spider phobia. However, the observed reduction in subjective fear did not translate into significant differences in objective behavioral or psychophysiological measures, indicating a need for a more comprehensive understanding of the complex interplay between subjective and objective outcomes in the treatment of phobias.

Future research should investigate the optimal dosage and frequency of VPA administration, the potential for variations in histone acetylation as a mediating factor, and the effects without retrieval. These considerations may contribute to a more comprehensive understanding of the mechanisms and therapeutic potential of VPA and HDAC inhibition in the treatment of specific phobias and potentially of other disorders treated with exposure therapy, with the potential to ultimately improve treatment efficacy and patient outcomes.

## Supporting information

Schicktanz et al. supplementary material Schicktanz et al. supplementary material

## Supplementary material

The supplementary material for this article can be found at http://doi.org/10.1017/S0033291725101475.
